# Immunohistochemical Evaluation of ALDH1 and Maspin in Oral Potentially Malignant Disorders and Oral Carcinoma

**DOI:** 10.3390/biomedicines14010079

**Published:** 2025-12-30

**Authors:** Bianca-Andreea Onofrei, Delia Gabriela Ciobanu Apostol, Mădălina-Gabriela Tanasă, Elena-Raluca Baciu, Cristina Popa, Ana Maria Sciuca, George Alexandru Maftei, Victor-Vlad Costan

**Affiliations:** Grigore T. Popa University of Medicine and Pharmacy Iasi, 700115 Iasi, Romania; bianca.onofrei@umfiasi.ro (B.-A.O.); madalina-gabriela.tanasa@umfiasi.ro (M.-G.T.); elena.baciu@umfiasi.ro (E.-R.B.); cristina.popa@umfiasi.ro (C.P.); ana.filioreanu@umfiasi.ro (A.M.S.); george-alexandru.maftei@umfiasi.ro (G.A.M.); victor.costan@umfiasi.ro (V.-V.C.)

**Keywords:** ALDH1, Maspin, oral leukoplakia, oral lichen planus, actinic cheilitis, oral squamous cell carcinoma

## Abstract

**Background/Objectives**: Oral potentially malignant disorders (OPMDs), including oral leukoplakia (OLK), oral lichen planus (OLP), and actinic cheilitis (AC), have varying risks of progression to oral squamous cell carcinoma (OSCC). Biomarkers such as aldehyde dehydrogenase 1 (ALDH1) and mammary serine protease inhibitor (Maspin) have shown potential for diagnostic and prognostic use in oral cancer. The present study aimed to evaluate the immunoexpression of aldehyde dehydrogenase 1, a cancer stem cell marker associated with aggressiveness, and the mammary serine protease inhibitor, a potential tumor suppressor, in OPMD and OSCC tissues. **Methods**: A retrospective analysis was performed on 145 biopsy specimens collected from January 2015 to January 2023, including normal epithelium, OPMDs (OLK, OLP, AC), and OSCC. ALDH1 and Maspin expression levels were evaluated using immunohistochemistry, considering both the percentage of positive cells and staining intensity. Statistical analyses were carried out using the Statistical Package for the Social Sciences (SPSS, version 29.0; IBM Corp., Chicago, IL, USA). **Results**: Normal oral epithelium showed no expression of ALDH1, whereas 40.6% of OPMDs and 44.4% of OSCC samples exhibited high cytoplasmic ALDH1 expression. Nuclear ALDH1 expression was elevated in 29.7% of OPMDs and 38.9% of OSCCs (*p* < 0.001). Nuclear Maspin expression was high in 95.2% of normal tissues, in 67.2% of OPMDs and in 55.6% of OSCCs (*p* < 0.001). Maspin showed strong nuclear and cytoplasmic expression in normal tissue, but its expression decreased in OPMDs and OSCCs, with statistically significant reductions in both compartments (*p* < 0.001). **Conclusions**: The results indicate that ALDH1 upregulation and Maspin downregulation are hallmark events in oral carcinogenesis. Their combined evaluation provides a powerful tool for assessing dysplastic severity and malignant transformation risk in OPMDs. Future studies on larger cohorts are needed to confirm the prognostic utility of this dual-marker model.

## 1. Introduction

Oral potentially malignant disorders (OPMDs) represent a broad and heterogeneous group of mucosal alterations that may precede the development of oral squamous cell carcinoma (OSCC). These lesions carry variable risks of malignant transformation, which makes it challenging to predict which cases will progress to cancer [1,2].

In order to gain a deeper understanding of the oncogenic potential of these lesions, a wide range of biological markers, such as aldehyde dehydrogenase 1 (ALDH1), mammary serine protease inhibitor (Maspin), differentiated embryonic chondrocyte 1 (DEC1), cluster of differentiation 44 (CD44), and E-cadherin, have been investigated [3,4,5]. Despite this, the role of ALDH1 and Maspin has been insufficiently analyzed, with only a limited number of studies addressing their expression and clinical relevance in this context.

ALDH1 is a cytosolic isoenzyme involved in detoxification processes, playing a critical role in the conversion of retinol into retinoic acid. This metabolite regulates gene expression, associated with cellular differentiation, apoptosis, and tumor progression, is also recognized as a specific marker for identifying stem cells [6,7]. In addition, retinoic acid plays an important role in modulating immune responses in inflammatory conditions [8,9]. Immunohistochemical evaluation of ALDH1 in OPMDs has so far been performed only in the epithelial compartment, where it serves as a marker of cancer stem cells [10].

Maspin, frequently expressed in normal epithelial tissues, acts as a tumor suppressor by enhancing cell adhesion and apoptosis, while reducing cell motility, angiogenesis, and pericellular proteolysis [11]. Its expression may be either upregulated or downregulated, depending on the tumor type, suggesting a possible prognostic value. Furthermore, Maspin is under active investigation for its therapeutic potential [12,13].

The present retrospective study was designed to investigate ALDH1 and Maspin expression and their associations with prognosis in a series of tissue samples collected from patients diagnosed with the three most common OPMDs in the north-eastern region of Romania, as well as from patients with OSCC. To achieve this aim, the following null hypothesis was proposed: there are no significant differences in ALDH1 and Maspin immunoscores between normal epithelium, OPMDs—oral leukoplakia (OLK), oral lichen planus (OLP), actinic cheilitis (AC), and OSCCs, nor any association with the degree of dysplasia or prognosis.

## 2. Materials and Methods

The present study followed the same research protocol and methodological approach as described in our previously published article [5].

### 2.1. Research Design

Biopsy tissue specimens collected between January 2015 and January 2023 were retrieved from the archives of the Pathology Department of “Sf. Spiridon” Emergency Clinical Hospital in Iași, Romania. The study received ethical approval from the Ethics Committees of both the “Sf. Spiridon” Emergency Clinical Hospital, Iași (No. 35/24 April 2023) and the Grigore T. Popa University of Medicine and Pharmacy, Iași (No. 320/5 June 2023). A total of 145 formalin-fixed, paraffin-embedded tissue samples were included in the study (Table 1) [5].

For the control group (normal epithelium specimens), adjacent non-inflamed tissue obtained during biopsy was used, while any inflamed or dysplastic areas were excluded. The diagnoses of OLK, OLP, AC, and OSCC were confirmed by the Pathology Department. To ensure diagnostic accuracy, all cases were independently reevaluated by two oral pathologists (D.G.C.A. and M.-G.T.). Inter-observer agreement was assessed using Cohen’s kappa statistic, which showed excellent agreement (κ > 0.80). Any disagreements between the two pathologists were resolved through discussion until consensus was reached.

All patients provided informed consent for the use of their tissues in research [5].

### 2.2. Immunohistochemical Analysis

Formalin-fixed, paraffin-embedded tissue blocks were cut with 4 μm thickness for immunohistochemistry (IHC) staining of ALDH1 and Maspin. The Mouse and Rabbit Specific HRP/DAB Detection IHC Kit, from Abcam (Cambridge, UK), was utilized according to the manufacturer’s protocol.

Deparaffinization of slides using xylene was performed, followed by rehydration in graded ethanol and distilled water. An antigen retrieval technique with citrate buffer, using a pressure boiler at 95 °C, for 40 min, was performed on all of the slides.

Endogenous peroxidase activity was blocked using a peroxidase-blocking reagent for 30 min. The slides were then washed with tris-buffered saline and incubated with primary antibodies from Abcam: rabbit recombinant monoclonal ALDH1 antibody (1:50) or rabbit polyclonal Maspin antibody (1:100), for 30 min, at 37 °C, in a humidifying chamber. Incubation with a secondary antibody conjugated with horseradish peroxidase (HRP) was performed and detected by chromogenic reaction of HRP.

Finally, the slides were counterstained with Mayer’s hematoxylin (5–10 min), dehydrated in three consecutive ethanol baths (1.5 min each), clarified with xylene (3 min), coverslipped, and observed under a microscope [5].

### 2.3. Antibodies

The commercial antibodies that were used are presented in Table 2.

**Figure 1:**
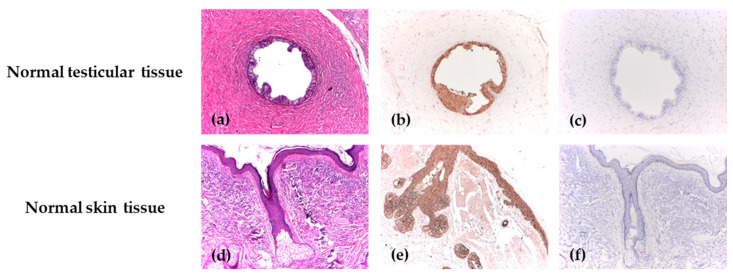
The positive and negative control reactions for ALDH1 and Maspin. (**a**) Deferent duct lined by a columnar epithelium, H&E, ×40; (**b**) Diffuse cytoplasmic positive immunoexpression of ALDH1 with strong intensity in the deferent duct, IHC, ×40; (**c**) Negative control of ALDH1 in the deferent duct, IHC, ×40; (**d**) Skin with normal morphology and a hair follicle with sebaceous gland, H&E, ×40; (**e**) Diffuse immunoexpression of Maspin, with strong intensity in normal epithelial tissue, IHC, ×40; (**f**) Negative control of Maspin in the skin tissue, IHC, ×40.

For each antibody, positive and negative control markers for the immunohistochemical reaction were used. For positive control reactions, two categories of normal tissues routinely available in the Pathology Department were selected and processed as dedicated control blocks. In accordance with the control tissues specified in the data sheet, the positive control for ALDH1 was the normal testicular tissue, where a specific cytoplasmic marker was identified, and for Maspin, the positive control was identified in normal skin tissue, where the antibody showed both nuclear and cytoplasmic markers. Skin and testicular tissues were chosen based on their consistent immunoreactivity profiles and confirmed absence of inflammatory, dysplastic, or other histopathological alterations. Immunohistochemical staining with ALDH1 was carried out similarly for the negative control, but tris buffer was used instead of the primary antibody. The primary antibody was omitted for the negative control in immunohistochemical staining with Maspin (Figure 1).

### 2.4. Evaluation of Score

The immunohistochemical evaluation of ALDH1 and Maspin expression was independently performed by two experienced pathologists by selecting five representative high-power fields. A semiquantitative analysis of antibody expression was conducted in epithelial cells at ×40 magnification. The proportion of positive cells was recorded and calculated as (the number of positive cells in a field/total number of cells) ×100. The evaluation included both the extent of staining (percentage of positive cells) and its intensity, and the final immunoscore was calculated differently for the two markers.

For ALDH1, the staining pattern was both cytoplasmic and nuclear. The percentage of positive cells (P) was categorized as 0% = score 0; <25% = score 1; 25–49% = score 2; 50–74% = score 3; and 75–100% = score 4. Intensity (I) scores for it were as follows: 0, no staining; 1, weak staining; 2, moderate staining; and 3, strong staining. The final immunoscore was obtained by multiplying the two scores (P × I) and ranged from 0 to 12. It was classified as low (≤1) or high (≥2) immunoexpression [14].

For Maspin, both nuclear and cytoplasmic expression were evaluated separately. The percentage of positive cells (P) was scored as follows: 0% = score 0; 1–5% = score 1; 6–50% = score 2; >50% = score 3. The staining intensity (I) for it was scored as follows: 0, no staining; 1, weak staining; 2, moderate staining; and 3, strong staining. The final immunoscore was obtained by summing the two scores (P + I) and ranged from 0 to 6. It was categorized as follows: 0, negative; 1 (1–2), low; 2 (3–4), moderate; and 3 (5–6), high immunoexpression [15].

Pathologists compared their results and discussed any discrepancies. This consensus approach was valuable in reaching a final score and minimizing bias. All slides were interpreted in comparison with adjacent areas of normal morphology used as controls for each patient.

### 2.5. Statistical Analysis

Descriptive statistical analyses were carried out using the Statistical Package for the Social Sciences (SPSS, version 29.0; IBM Corp., Chicago, IL, USA). As the data did not follow a normal distribution, non-parametric tests were applied. Differences between groups were assessed using the Pearson Chi-squared and Kruskal–Wallis tests, with statistical significance set at *p* < 0.05.

## 3. Results

### 3.1. Comparative Analysis of ALDH1 and Maspin in Normal Epithelium, OPMD and OSCC

**Figure 2:**
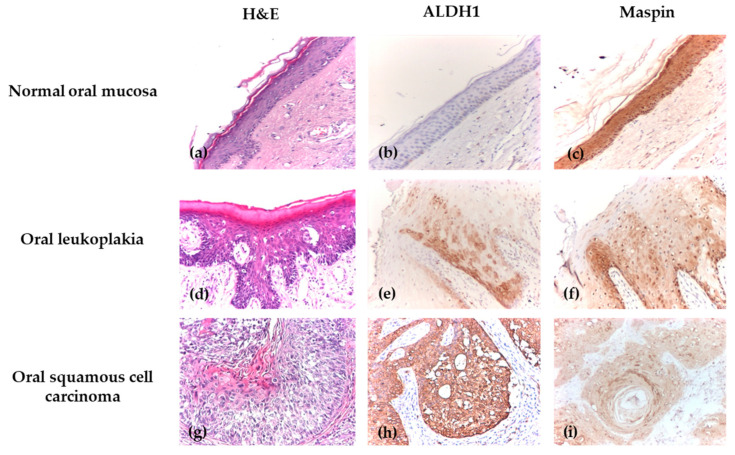
Immunohistochemical analysis of normal epithelium, OLK, OSCC. (**a**) Normal oral mucosa, H&E, ×100; (**b**) Normal oral mucosa with negative cytoplasmic and nuclear ALDH1 immunoexpression, IHC, ×100; (**c**) Normal oral mucosa with positive Maspin immunoexpression, showing strong staining cytoplasmic and nuclear intensity, IHC, ×100; (**d**) Oral leukoplakia with moderate dysplasia, H&E, ×100; (**e**) OLK with mild dysplasia, with positive cytoplasmic and nuclear ALDH1 immunoexpression, showing moderate intensity, IHC, ×100; (**f**) OLK with mild dysplasia, showing positive Maspin immunoexpression with extensive intraepithelial distribution, weak cytoplasmic and nuclear intensity, IHC, ×100; (**g**) Well-differentiated oral squamous cell carcinoma, H&E, ×100; (**h**) Well-differentiated OSCC with positive ALDH1 immunoexpression and extensive intraepithelial distribution, showing strong cytoplasmic and nuclear intensity, IHC, ×100; (**i**) Well-differentiated OSCC with positive cytoplasmic and nuclear Maspin immunoexpression, showing weak intensity and extensive distribution throughout the tumor area, IHC, ×100.

The immunoexpression profiles of ALDH1 and Maspin revealed fundamentally opposite patterns in OPMDs compared to normal epithelium, suggesting their potential involvement in early oral carcinogenesis. Representative morphological features on hematoxylin–eosin (H&E) staining and immunohistochemical expression patterns of ALDH1 and Maspin in normal oral epithelium and oral lesions are illustrated in Figure 2.

ALDH1 emerged as a marker of malignant potential, with its expression upregulated in OPMDs. This was evident both in the cytoplasm and nucleus, where 40.6% and 29.7% of OPMD cases, respectively, contrasted with its complete absence in normal tissue.

Conversely, Maspin expression was downregulated in OPMDs, suggesting a loss of its protective role. While high immunoexpression was nearly universal in normal epithelium (95.2% of cases), it was reduced in OPMDs, with only 60.9% of cases showing high cytoplasmic immunoexpression and 67.2% showing high nuclear immunoexpression.

The contrasting immunoexpression profiles of ALDH1 and Maspin observed in OPMDs became even more evident in malignant lesions, underscoring their potential involvement in oral cancer progression. ALDH1 exhibited an upregulation in oral carcinomas, supporting its association with more aggressive tumor phenotypes. Cytoplasmic expression was increased, with 44.4% of malignant cases demonstrating high levels, in contrast to its complete absence in normal tissue. A similar trend was observed for nuclear expression, with 38.9% of malignancies exhibiting high levels. In contrast, Maspin showed a downregulation in malignant lesions, suggesting a gradual loss of its tumor-suppressive role. High cytoplasmic immunoexpression was maintained in only 50.0% of malignant cases compared with 95.2% of normal samples, while nuclear expression was reduced to 55.6% in malignancies versus 95.2% in normal epithelium (Table 3).

The quantitative mean expression levels for all markers and locations consistently and significantly reflected these trends, further solidifying the contrast between normal tissue, OPMDs and OSCCs. These data are summarized in Table 3 and Table 4.

### 3.2. Comparative Analysis of ALDH1 and Maspin Immunoexpression in Relation to Dysplasia Grade

**Figure 3:**
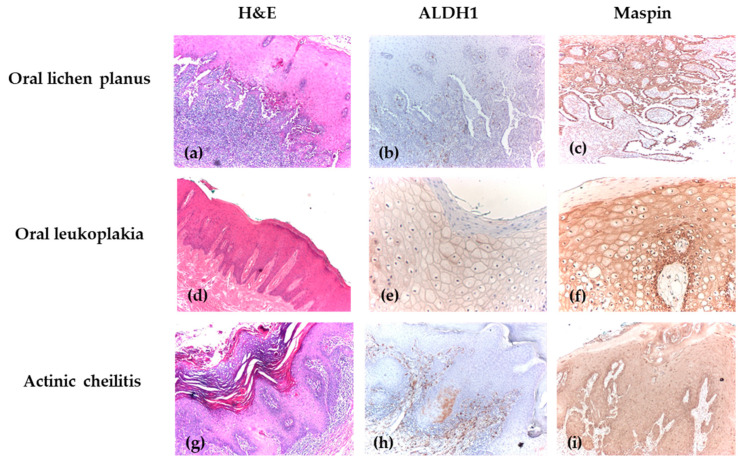
Immunohistochemical analysis of OPMDs. (**a**) OLP without dysplasia, showing Civatte cells, H&E, ×40; (**b**) OLP with negative ALDH1 immunoexpression, IHC, ×40; (**c**) OLP with positive cytoplasmic and nuclear Maspin immunoexpression, showing strong intensity and extensive intraepithelial distribution, IHC, ×40; (**d**) OLK with mild dysplasia affecting one-third of the epithelial thickness, with pseudoepitheliomatous hyperplasia of the epithelium accompanied by parakeratosis at its surface, associated with reduced lympho-plasmacytic inflammatory infiltrate, H&E, ×40; (**e**) OLK with positive cytoplasmic ALDH1 immunoexpression, showing weak intensity and extensive intraepithelial distribution, IHC, ×100; (**f**) OLK with positive cytoplasmic Maspin immunoexpression (strong intensity) and nuclear Maspin immunoexpression (moderate intensity), showing extensive intraepithelial distribution, IHC, ×100; (**g**) AC with severe dysplasia, H&E, ×40; (**h**) AC with positive cytoplasmic ALDH1 immunoexpression (weak intensity) and negative nuclear expression, IHC, ×40; (**i**) AC with positive Maspin immunoexpression, showing weak intensity and extensive intraepithelial distribution, IHC, ×40.

The expression patterns of ALDH1 and Maspin showed distinct correlations with the severity of epithelial dysplasia, revealing their potential roles in oral carcinogenesis progression. Morphological characteristics on H&E staining and immunohistochemical profiles of ALDH1 and Maspin across different grades of oral epithelial dysplasia are presented in Figure 3.

Cytoplasmic ALDH1 expression appeared to increase with dysplasia severity, peaking in grades 1–2, where 52.6–57.9% of cases showed high immunoexpression compared to only 10.5% in grade 0. In contrast, nuclear ALDH1 expression showed no significant variation across grades, with high immunoexpression observed in 42.1–42.9% of cases in grades 2–3 compared to only 10.5% in grade 0. Maspin expression showed a progressive loss with increasing dysplasia grade. Both cytoplasmic and nuclear Maspin expression were significantly higher in grade 0 dysplasia (84.2% and 89.5% high expression, respectively) and decreased markedly in higher grades, with only 47.4–57.1% of cases showing high expression in grades 2–3 (Table 5 and Table 6).

## 4. Discussion

The present study demonstrated divergent expression patterns of ALDH1 and Maspin across normal epithelium, OPMDs and OSCCs, thereby leading to the rejection of the null hypothesis.

In our study, ALDH1 expression showed significant differences between normal oral epithelium and both premalignant and malignant lesions; however, it did not discriminate between degrees of epithelial dysplasia. Nuclear ALDH1 has been rarely described in the literature, with only one prior report by Zisis et al. [16], who documented nuclear staining in oral leukoplakia and squamous cell carcinoma. Differences in antibody clones between that study and ours (as specified in the Methods) may partially explain the variability in nuclear localization reported across studies.

The biological basis for nuclear ALDH1 expression remains incompletely understood. ALDH1 catalyzes the oxidation of retinaldehyde to retinoic acid (RA), a key regulator of cellular differentiation acting through nuclear RAR/RXR receptors [17]. One possibility is that RA synthesis may occur adjacent to—or within—the nuclear compartment, enabling rapid ligand–receptor interactions and localized transcriptional regulation. Nuclear ALDH1 may therefore mark a specific functional state rather than a broad phenotypic shift.

The lack of statistical significance between dysplasia grades likely reflects underlying biological factors. These include: (1) the compartment-specific roles of cancer stem cell subpopulations, of which nuclear ALDH1-positive cells may represent only a minority; (2) metabolic heterogeneity across dysplastic lesions; and (3) the predominantly cytoplasmic function of ALDH1, which may yield more consistent changes than the rarer nuclear signal. Consequently, nuclear ALDH1 staining alone may not be sufficiently sensitive to differentiate between histologic grades of premalignant lesions, despite its association with malignant transformation.

The trends observed in our study are consistent with the findings of Dhumal et al. [18], who examined ALDH1 expression immunohistochemically in 79 cases, including 25 normal oral mucosa samples, 30 OPMDs, and 24 OSCC cases with and without nodal metastases. ALDH1 was absent in all normal epithelium samples, while in OPMDs with dysplasia, a significantly higher cytoplasmic immunoexpression was noted compared to those without dysplasia (*p* < 0.05). Although expression levels were higher in high-risk dysplasias compared to low-risk ones, these differences did not reach statistical significance in all cases. In OSCC, ALDH1 was detected in 29.1% of cases, mainly in invasive tumor cells and the overlying dysplastic epithelium. Importantly, the mean immunohistochemical score was higher in tumors with nodal metastases than in those without, suggesting a potential association between ALDH1 expression and tumor aggressiveness. Similar results were reported by Abdulmajeed et al. [19], who analyzed the immunoexpression of ALDH1, CD24, and CD44 markers in an initial batch of 107 biopsies, aiming to establish clinically relevant immunohistochemical scores, later applied to an independent validation cohort of 278 biopsies. Their findings confirmed a significantly increased intensity of ALDH1 immunostaining in OSCC compared to normal epithelium, and the immunoexpression of this marker correlated with the severity of epithelial changes.

Thankappan et al. [14] provided evidence that stem cell markers, particularly ALDH1, play a significant role in assessing malignant transformation risk in oral epithelial dysplasias (OED). In their cohort, ALDH1 expression was observed in over 70% of dysplasia cases but only in 10% of normal mucosa, with higher immunoscores in moderate (6.30 ± 4.36) and severe (5.27 ± 3.05) dysplasias (*p* < 0.001). Notably, expression extended from the upper layers in mild cases to the basal layer in advanced dysplasias, supporting its involvement in disease progression. Moreover, co-expression with CD44, SOX2, and OCT4 identified a subpopulation of cells with enhanced malignant potential, highlighting the prognostic value of combined stem cell marker analysis.

Maspin exhibits dual, localization-dependent functions with important diagnostic and prognostic implications. Nuclear Maspin generally acts as a tumor suppressor through transcriptional regulation and HDAC1 inhibition, whereas cytoplasmic Maspin may promote invasion via EMT–related signaling pathways. In oral premalignant lesions, Maspin is typically expressed in both compartments, reflecting its role in tissue homeostasis. During progression to OSCC, Maspin expression—particularly at the invasive front—declines, and reduced Maspin levels correlate with poorer differentiation and increased metastatic risk. High Maspin expression is associated with a more favorable prognosis (pN0), whereas low or absent expression predicts lymph node metastasis and shorter disease-free survival [12].

However, the prognostic role of Maspin remains complex and context-dependent. Numerous studies have investigated its value in OSCC and other cancers, with conclusions ranging from favorable to unfavorable, largely depending on the tumor context and its intracellular localization [20,21,22]. In head and neck squamous cell carcinoma, for instance, increased Maspin expression has been correlated with longer survival and the absence of nodal metastases [23]. A critical factor appears to be its subcellular localization: marked nuclear expression is often associated with a favorable prognosis, while exclusive cytoplasmic expression is correlated with unfavorable prognostic factors [24,25]. This supports the idea that nuclear localization of Maspin may reflect its biologically active form responsible for tumor-suppressor activity, while cytoplasmic localization might indicate a functionally inactive form [26]. The patterns observed in our study align with this model, showing that the progressive reduction in Maspin—highly expressed in both the cytoplasm and nucleus of normal epithelium, but gradually lost in OPMDs and further reduced in OSCCs—reflects a diminishing tumor-suppressive capacity during malignant progression. Overall, these fundings support Maspin as a useful prognostic biomarker, and understanding the mechanisms governing its subcellular localization may provide future therapeutic opportunities.

These biomarkers are not yet part of standard clinical workflows for oral premalignant lesions or squamous cell carcinoma, but their biological functions suggest that they could be useful. ALDH1, widely recognized as a cancer stem cell marker, has been associated with tumor initiation, progression, metastatic potential, and resistance to therapy [27], while Maspin demonstrates variable expression during the transition from premalignant to malignant states, reflecting changes in tissue remodeling and tumor suppression activity [12]. These complementary mechanisms provide a rationale for evaluating the two markers in concert rather than independently. In our context, a dual ALDH1–Maspin immunohistochemical model may offer a more nuanced stratification of malignant transformation risk. High ALDH1 coupled with low Maspin expression appears to identify lesions with a more aggressive biological profile, supporting intensified patient surveillance and potentially earlier intervention. Conversely, lesions characterized by low ALDH1 and high Maspin expression may correspond to a low-risk profile, suitable for conservative treatment and standard follow-up. Such a biomarker-based approach, when integrated with established clinicopathological indicators—including lesion size, grade, and lymph node status—could enhance risk stratification algorithms and contribute to more individualized treatment pathways.

Although the relatively small cohort size and the absence of extensive correlations with clinical outcome parameters constitute limitations of this study, the obtained results underscore the value of immunohistochemical investigations in the biological characterization of oral lesions. In addition, the unequal distribution of cases across study groups (the smaller number of OSCC samples) may reduce the statistical software’s ability to detect more subtle differences in biomarker expression. Furthermore, the evaluation according to tumor differentiation and TNM stage would further enhance the clinical interpretation of our results. Future studies on larger, prospectively designed cohorts, incorporating long-term follow-up data, are essential to validate the prognostic utility of this antagonistic expression model and to definitively establish its role in clinical decision-making algorithms. At this stage, ROC curve analysis falls beyond the scope of our retrospective dataset; however, we acknowledge its importance and will consider integrating ROC-based validation in future studies with larger cohorts.

## 5. Conclusions

The present study revealed significant differences between ALDH1 and Maspin markers in OPMDs and in OSCC, outlining an antagonistic expression pattern.

The comparative analysis of the two markers offers a complementary perspective on lesion evolution: ALDH1 signals the presence of cellular subpopulations with increased malignant potential, while the decrease in Maspin reflects the loss of proliferation and invasion control mechanisms. Therefore, the simultaneous evaluation of ALDH1 and Maspin expression allows for a more nuanced approach to oncological risk, surpassing the limitations of traditional morphological diagnosis.

The integration of the two markers into a dual molecular evaluation model can significantly contribute to improving early diagnosis and prognostic strategies in oral premalignant and malignant lesions, supporting the clinical utility of immunohistochemical investigations in pathological practice.

## Figures and Tables

**Table 1:** Clinicopathological characteristics of the study groups.

Group	Diagnosis	Number				
Group 0 control group	Normal epithelium	63				
Group 1	Oral potentially malignant disorders		Dysplasia grade			
			0	1	2	3
	OLK	24	5	7	12	-
	OLP	13	13	-	-	-
	AC	27	1	12	7	7
		Total = 64				
Group 2	Oral squamous cell carcinoma	18				
		Total = 145				

**Table 2:** Specifications of primary antibodies and IHC protocols.

Antibody	Clone	pH	Class	Dilution	Expression
ALDH1 (Abcam, Cambridge, UK)	EP1933Y	6	mouse, monoclonal	1:50	cytoplasmic
Maspin (Abcam, Cambridge, UK)	Polyclonal	6	mouse, polyclonal	1:100	nuclear cytoplasmic

**Table 3:** Comparative ALDH1 and Maspin immunoexpression profiles in the study groups.

			Study Groups						*p*-Value
			Normal Epithelium		OPMD		OSCC		
			N	%	N	%	N	%	
ALDH1	Cytoplasmic	Low	63	100.0%	38	59.4%	10	55.6%	<0.001 *
		High	-	-	26	40.6%	8	44.4%	
	Nuclear	Low	63	100.0%	45	70.3%	11	61.1%	<0.001 *
		High	-	-	19	29.7%	7	38.9%	
Maspin	Cytoplasmic	Low	-	-	-	-	1	5.6%	<0.001 *
		Moderate	3	4.8%	25	39.1%	8	44.4%	
		High	60	95.2%	39	60.9%	9	50.0%	
	Nuclear	Negative	-	-	1	1.6%	1	5.6%	<0.001 *
		Low	-	-	-	-	1	5.6%	
		Moderate	3	4.8%	20	31.3%	6	33.3%	
		High	60	95.2%	43	67.2%	10	55.6%	

* Pearson Chi-squared test, statistical significance, *p* < 0.05.

**Table 4:** Intergroup comparison of ALDH1 and Maspin immunoexpression levels across normal epithelium, OPMD and OSCC.

		Study Groups (Mean ± SD)			*p*-Value
		Normal Epithelium	OPMD	OSCC	
ALDH1	Cytoplasmic	0.00 ± 0.000	1.17 ± 1.638	1.50 ± 2.834	<0.001 *
	Nuclear	0.00 ± 0.000	1.00 ± 1.799	1.39 ± 2.852	<0.001 *
Maspin	Cytoplasmic	7.90 ± 1.729	5.34 ± 2.169	4.94 ± 2.363	<0.001 *
	Nuclear	7.95 ± 1.717	5.91 ± 2.505	5.28 ± 2.886	<0.001 *

* Kruskal–Wallis test; statistical significance, *p* < 0.05; SD—standard deviation.

**Table 5:** Comparative ALDH1 and Maspin immunoexpression profiles in relation to dysplasia grade.

			Dysplasia Grade								*p*-Value
			0		1		2		3		
			N	%	N	%	N	%	N	%	
ALDH1	Cytoplasmic	Low	17	89.5%	9	47.4%	8	42.1%	4	57.1%	0.014 *
		High	2	10.5%	10	52.6%	11	57.9%	3	42.9%	
	Nuclear	Low	17	89.5%	13	68.4%	11	57.9%	4	57.1%	0.147
		High	2	10.5%	6	31.6%	8	42.1%	3	42.9%	
Maspin	Cytoplasmic	Moderate	3	15.8%	9	47.4%	10	52.6%	3	42.9%	0.094
		High	16	84.2%	10	52.6%	9	47.4%	4	57.1%	
	Nuclear	Negative	-	-	1	5.3%	-	-	-	-	0.225
		Moderate	2	10.5%	7	36.8%	8	42.1%	3	42.9%	
		High	17	89.5%	11	57.9%	11	57.9%	4	57.1%	

* Pearson Chi-squared test, statistical significance, *p* < 0.05.

**Table 6:** Intergroup comparison of ALDH1 and Maspin immunoexpression levels in relation to dysplasia grade.

		Dysplasia Grade (Mean ± SD)				*p*-Value
		0	1	2	3	
ALDH1	Cytoplasmic	0.21 ± 0.631	1.79 ± 2.043	1.58 ± 1.644	1.00 ± 1.291	0.010 *
	Nuclear	0.42 ± 1.427	1.42 ± 2.341	1.16 ± 1.642	1.00 ± 1.291	0.216
Maspin	Cytoplasmic	6.79 ± 2.200	4.74 ± 1.821	4.58 ± 1.835	5.14 ± 2.268	0.010 *
	Nuclear	7.74 ± 2.077	5.05 ± 2.460	5.21 ± 2.200	5.14 ± 2.268	0.002 *

* Kruskal–Wallis Test; statistical significance, *p* < 0.05; SD—standard deviation.

## Data Availability

The original contributions presented in this study are included in the article. Further inquiries can be directed to the corresponding author.

## Abbreviations

The following abbreviations are used in this manuscript:

ALDH1	Aldehyde dehydrogenase 1
CD44	Cluster of differentiation 44
DEC1	Differentiated embryonic chondrocyte 1
H&E	Hematoxylin–eosin
HRP	Horseradish peroxidase
I	Intensity of Positivity
IHC	Immunohistochemistry
Maspin	Mammary serine protease inhibitor
OED	Oral epithelial dysplasias
OPMDs	Oral potentially malignant disorders
OSCC	Oral squamous cell carcinoma
P	Extent of positivity
SPSS	Statistical Package for the Social Sciences
